# Morphological Heterogeneity in Pancreatic Cancer Reflects Structural and Functional Divergence

**DOI:** 10.3390/cancers13040895

**Published:** 2021-02-20

**Authors:** Petra Sántha, Daniela Lenggenhager, Anette Finstadsveen, Linda Dorg, Kristin Tøndel, Manoj Amrutkar, Ivar P. Gladhaug, Caroline Verbeke

**Affiliations:** 1 Department of Pathology, Oslo University Hospital, Rikshospitalet, 0424 Oslo, Norway; santha.petra@gmail.com (P.S.); uxvene@ous-hf.no (A.F.); manoj.amrutkar@medisin.uio.no (M.A.); 2 Department of Pathology and Molecular Pathology, University Hospital Zürich, University of Zürich, 8091 Zürich, Switzerland; Daniela.Lenggenhager@usz.ch; 3 Department of Pathology, Institute of Clinical Medicine, University of Oslo, 0316 Oslo, Norway; l.t.dorg@medisin.uio.no; 4 Department of Data Science, Faculty of Science and Technology, Norwegian University of Life Sciences, 1430 Ås, Norway; kristin.tondel@nmbu.no; 5 Department of Pharmacology, Institute of Clinical Medicine, University of Oslo, 0316 Oslo, Norway; 6 Department of Hepato-Pancreato-Biliary Surgery, Oslo University Hospital, Rikshospitalet, 0424 Oslo, Norway; i.p.gladhaug@medisin.uio.no; 7 Department of Hepato-Pancreato-Biliary Surgery, Institute of Clinical Medicine, University of Oslo, 0316 Oslo, Norway

**Keywords:** human pancreatic ductal adenocarcinoma, morphology, pathology, heterogeneity, stroma, extracellular matrix

## Abstract

**Simple Summary:**

Pancreatic cancer has a poor prognosis, which is largely due to resistance to treatment. Tumor heterogeneity is a known cause for treatment failure and has been studied at the molecular level. Morphological heterogeneity is common but has not been investigated, despite the fact that pathology examination is an integral part of clinical diagnostics. This study assessed whether morphological heterogeneity reflects structural and functional diversity in key cancer biological processes. Using archival tissues from resected pancreatic cancer, we selected four common and distinct morphological phenotypes and demonstrated that these differed significantly for a panel of 26 structural and functional features of the cancer-cell and stromal compartments. The strong link between these features and morphological phenotypes allowed prediction of the latter based on the results for the panel of features. The findings of this study indicate that morphological heterogeneity reflects biological diversity and that its assessment may potentially provide clinically relevant information.

**Abstract:**

Inter- and intratumor heterogeneity is an important cause of treatment failure. In human pancreatic cancer (PC), heterogeneity has been investigated almost exclusively at the genomic and transcriptional level. Morphological heterogeneity, though prominent and potentially easily assessable in clinical practice, remains unexplored. This proof-of-concept study aims at demonstrating that morphological heterogeneity reflects structural and functional divergence. From the wide morphological spectrum of conventional PC, four common and distinctive patterns were investigated in 233 foci from 39 surgical specimens. Twenty-six features involved in key biological processes in PC were analyzed (immuno-)histochemically and morphometrically: cancer cell proliferation (Ki67) and migration (collagen fiber alignment, MMP14), cancer stem cells (CD44, CD133, ALDH1), amount, composition and spatial arrangement of extracellular matrix (epithelial proximity, total collagen, collagen I and III, fibronectin, hyaluronan), cancer-associated fibroblasts (density, αSMA), and cancer-stroma interactions (integrins α2, α5, α1; caveolin-1). All features differed significantly between at least two of the patterns. Stromal and cancer-cell-related features co-varied with morphology and allowed prediction of the morphological pattern. In conclusion, morphological heterogeneity in the cancer-cell and stromal compartments of PC correlates with structural and functional diversity. As such, histopathology has the potential to inform on the operationality of key biological processes in individual tumors.

## 1. Introduction

Ductal adenocarcinoma of the pancreas, often referred to as pancreatic cancer (PC), has a dismal prognosis with an overall five-year survival of less than 7% [1,2]. In addition to late diagnosis, limited efficacy of current treatment is the main reason for the poor prognosis. Precision medicine holds the promise of improved outcome through treatment that is tailored to intrinsic properties of the individual tumor. This approach requires a classification system with well-defined tumor subtypes of a cancer entity, as it is established, for example, for breast cancer. Several classification systems have been proposed for PC, which are based on gene expression profiling [3,4,5,6]. However, the latter is not without its practical obstacles, including the need for RNA of sufficient quantity and quality. In addition, there is the unresolved problem of intratumor heterogeneity.

In contrast to active research regarding a molecular taxonomy for PC, morphological variation in PC has received little if any attention, except for a few rare subtypes of PC with unusual morphological features—for example, medullary and colloid carcinomas which are associated with microsatellite instability [7,8]. While these subtypes account for less than 10% of PC, the vast majority of tumors constitute one large group that is denoted as PC not otherwise specified (NOS). In this group, morphological variation is common but considered irrelevant and largely left without further characterization [9]. Only recently, a few attempts have been made to correlate genotype or transcriptional profile with a small number of broad histological categories that encompass a wide range of morphologies of PC [10]. Despite these initial investigations, the relationship between morphological heterogeneity and biological diversity in PC is far from understood. In this study, we aimed at providing proof of concept that differences in morphology are associated with structural and functional divergence in biological processes deemed to be relevant in terms of PC biology and/or clinical outcome. Four common but not previously described morphological patterns of PC were selected from the wide spectrum of morphologies observed in PC NOS [11]. Human PC tissues containing such patterns were investigated with (immuno-)histochemistry and morphometry in order to characterize them for a panel of 26 features that are known to play a role in key aspects of PC biology, including cancer cell proliferation (Ki67) and migration (collagen fiber alignment, matrix metalloproteinase 14), cancer stem cells (CD44, CD133, aldehyde dehydrogenase 1), amount, composition and spatial arrangement of the extracellular matrix (epithelial proximity, total collagen, collagen I and III, fibronectin, hyaluronan), cancer-associated fibroblasts (density, α-smooth muscle actin), and cancer-stroma interactions (integrins α2, α5, β1, caveolin-1). All of the non-structural features analyzed in this study are part of recently compiled comprehensive repertoires of compartment-specific genes in PC [3,12]. The biological roles and clinical relevance of the selected features are summarized in Table 1.

## 2. Results

### 2.1. Tumor Patterns

**Figure 1:**
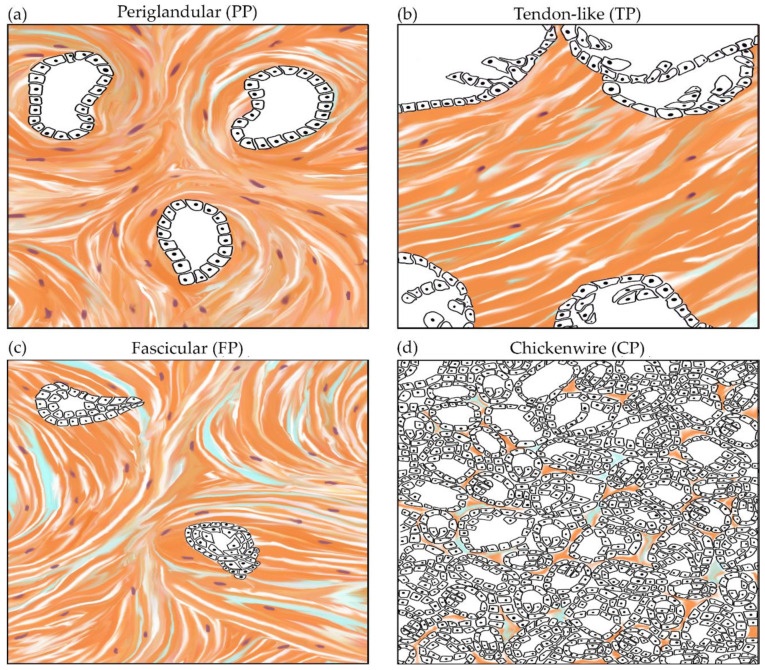
Four patterns of pancreatic cancer with distinctive morphological features of the cancer-cell and stromal compartments. (**a**) The periglandular pattern (PP) is characterized by medium-sized simple glands surrounded by a moderate amount of stroma that encircles the tumor glands. (**b**) In the tendon-like pattern (TP), the large cystic-papillary tumor glands are embedded in a vast and dense fibrous stroma. (**c**) The fascicular pattern (FP) typically contains medium-sized tumor glands that are often angulated and lie in a highly cellular, whorled stroma. (**d**) In the chickenwire pattern (CP), tumor glands are small and lie densely packed with little intervening stroma.

From the wide range of morphologies that can be encountered in PC NOS, four commonly seen patterns were selected and defined by distinctive features that are readily assessable on hematoxylin and eosin (H&E) staining in both the cancer-cell and stromal compartments, as shown in Figure 1 and illustrated in detail in Figure S1. Reflecting the morphology of their stroma, the patterns were coined periglandular (PP), tendon-like (TP), fascicular (FP), and chickenwire (CP). These patterns are not described previously, but the cancer cell-component of TP is reminiscent of the cystic papillary variant mentioned in the World Health Organisation (WHO) classification [7]. The four patterns are characterized by glandular tumor growth and are therefore well differentiated [96].

### 2.2. Study Series

**Figure 2:**
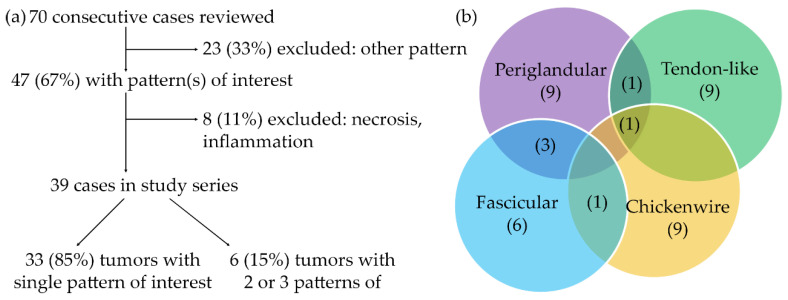
Case selection process and study series composition. (**a**) Flow chart depicting the selection process resulting in the study series. (**b**) Venn-diagram illustrating the number of tumors with a single, two or three different patterns.

Of the 70 consecutive, treatment-naïve cases that were reviewed, 47 (67%) contained a pattern of interest, either exclusively or in combination with other morphologies that are not investigated in this study. Following exclusion of eight cases because of necrosis or marked inflammation which alter tumor morphology, 39 cases were included in the study series, of which 33 contained one of the four patterns that were analyzed in this study and six cases contained two or three different patterns (Figure 2). In each case of the series, at least five and at most eight regions of interest (ROIs) representative of a particular pattern were analyzed for the panel of features, except in two cases with PP, in which only three ROIs were available for analysis, and in one case of FP with only four ROIs. Overall, investigation of the panel of 26 features in a total number of 233 ROIs (PP: 68; TP: 60; FP: 50; CP: 55) engendered 816 sections with the various stains. Clinicopathological features of the study series are shown in Table S1.

### 2.3. Cancer-Cell and Stromal Features

**Figure 3:**
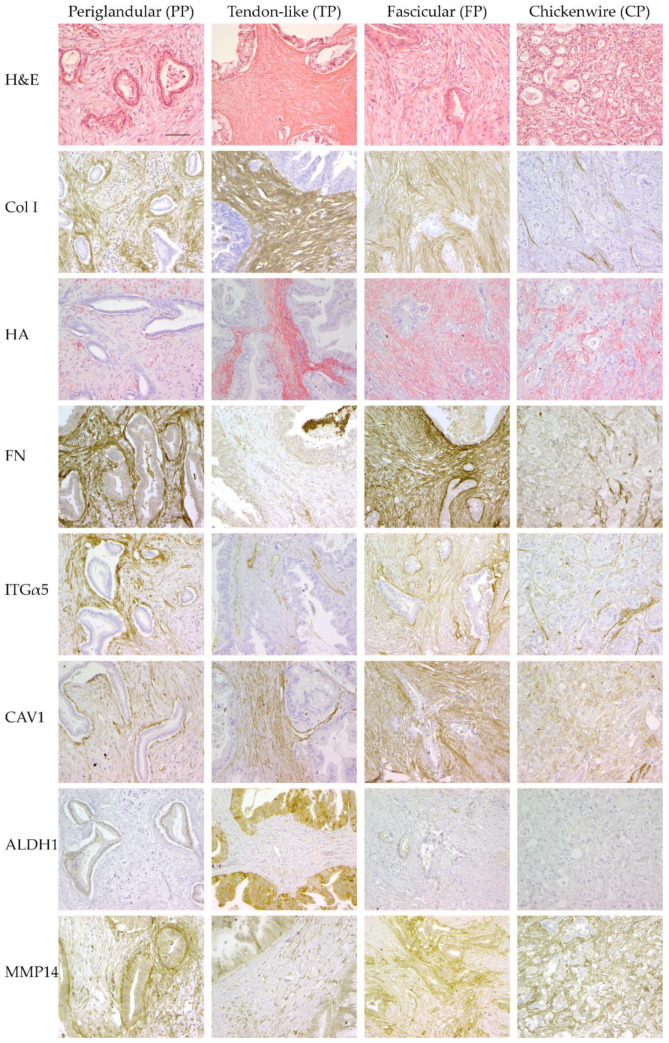
Divergence of stromal and cancer cell-related features in the four pancreatic cancer patterns. Representative microphotographs of hematoxylin and eosin (H&E) staining and (immuno-)histochemistry for selected features (200x; scale bar = 100 μm) are shown. ALDH1, aldehyde dehydrogenase 1; CAV1, caveolin-1; Col I, collagen I; HA, hyaluronan; H&E, hematoxylin and eosin; FN, fibronectin; ITGα5, integrinα5; MMP14, matrix metalloproteinase 14.

**Figure 4:**
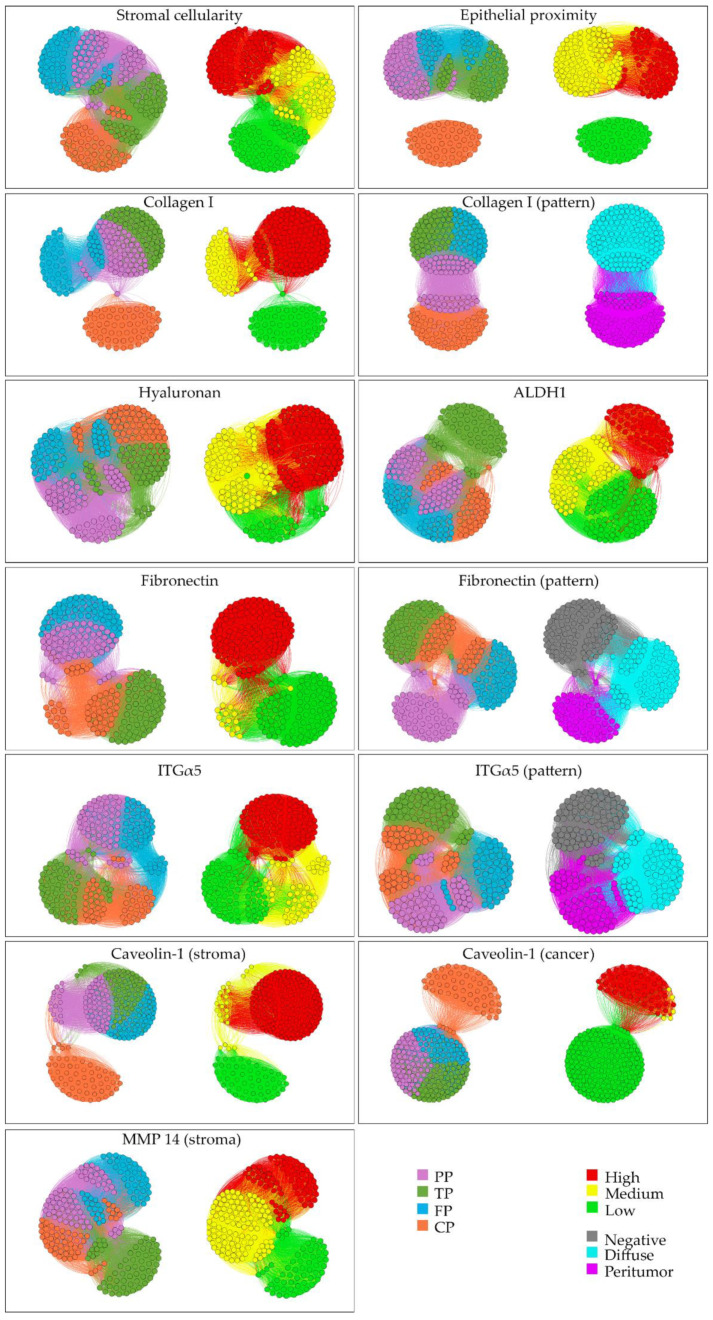
Complex network visualization of cancer-cell-related and stromal features in the four tumor patterns. For a particular feature, paired Gephi-generated networks visualize the four morphological patterns (left) and the results for that feature (right). Every dot in the network represents a single ROI, and the length of the connecting lines represents the similarity between the connected nodes (*n* = 233). Absence of connecting lines between dots (e.g., in Gephi for epithelial proximity) indicates the lack of similarity. For each feature, the *p*-value reflecting the difference between the patterns is stated in Table 2.

Significant differences between at least two of the four patterns were observed for all features. The main findings are described below and illustrated in Figure 3. Network visualization is shown in Figure 4. The results of the statistical analysis and all individual data are detailed in Table 2 and Table S2, respectively.

#### 2.3.1. Cancer-Cell Features

Proliferative activity, assessed with the Ki67 index, in the entire series was close to identical with that observed by others (mean: 27%) [97,98]. However, it differed significantly between all four patterns and was found to be highest in CP (42%), followed in descending order by FP (31%), PP (23%), and TP (13%) (*p* < 0.001).

Three cancer stem cell (CSC) markers were studied. As reported by others, cytoplasmic immunostaining for CD44 was found in the majority of cases (83%) [30,34,35,39,40,51], but expression was significantly less frequent and at a lower level in PP than in the other patterns (*p* ≤ 0.007). Similarly, expression of CD133, typically limited to the apical cell membrane, was in line with previous reports for the overall series [39,40,48,50,51] but differed between the patterns. It was significantly higher in PP and FP (36% and 46% high, respectively) than in TP and CP (93% and 100% low or negative, respectively) (*p* < 0.001) Cytoplasmic ALDH1-expression was significantly higher in TP (87% high) than in the three other patterns (0–2% high) (*p* < 0.001) [13,17,18,51,99] (Figure 3 and Figure 4). There was no co-variation between CD44, CD133 and ALDH1, either overall or for each of the patterns individually.

Expression of integrin (ITG) α2 and β1, which together form the cellular receptor for collagen I, was significantly lower in PP than in the other patterns (*p* < 0.001). Matrix metalloproteinase 14 (MMP14), which through its ECM-degrading effect promotes cancer cell migration [58], was expressed at significantly different levels by cancer cells in the four patterns: highest in FP (58%), followed by CP (20%) and PP (13%), and TP (2%) (*p* ≤ 0.037), respectively (Figure 3 and Figure 4). Caveolin-1 (CAV1) can be expressed by both cancer cells and cancer-associated fibroblasts (CAFs) and has multifarious effects, including the promotion of proliferation, invasion and chemoresistance [24,25]. CAV1-expression was significantly higher in cancer cells of CP (82%) than in the other patterns (0%; *p* < 0.001) (Figure 3 and Figure 4).

#### 2.3.2. Stromal Features

For the purpose of this study, the stroma that lies immediately adjacent to the cancer cells (that is, within a 200x high-power field) was investigated rather than the bulk stroma, as it is the former that plays a pivotal role in the cancer cell biology [19,100,101]. Among stromal features, epithelial proximity, a proxy for the dispersedness of the cancer glands and the amount of intervening stroma, differed significantly between all patterns (*p* < 0.001), being highest in TP, followed in descending order by FP, PP and CP. The total amount of collagen was highest in TP and moderate in PP (*p* < 0.001), but similarly low in FP and CP. Collagen I deposition in PP and CP was peritumoral—that is, the fibers formed a sheath that encircled the tumor glands, while it was diffuse in TP and FP (Figure 3 and Figure 4). Results for collagen III were similar, though immunostaining levels were overall lower than for collagen I. Collagen fiber alignment was highest in TP (*p* ≤ 0.001) and moderate in PP (*p* ≤ 0.011) but low in FP and CP. Fibronectin content was highest in FP, moderate in PP, low in CP and absent in almost all TP (*p* < 0.001). Fibronectin deposition was mainly peritumoral in PP (75%) and diffuse in FP (100%), where it was organized in the sweeping bundles that characterize the whorled stromal appearance in this pattern (Figure 3 and Figure 4).

Results for the entire study series regarding hyaluronan were highly similar with those reported by others (high-level expression in 55% [33,63,82,102]). However, levels differed significantly between patterns: they were highest in TP and CP (*p* < 0.001), and lowest in PP (*p* ≤ 0.002) (Figure 3 and Figure 4). In all patterns, the vast majority of stromal cells with fibroblast morphology were αSMA-positive, confirming their nature as activated CAFs [103]. Stromal cell density was highest in FP, lowest in TP and CP (*p* ≤ 0.008).

The ratio between the number of αSMA + CAFs and amount of collagen, the so-called activated stroma ratio has been used to characterize and categorize the stroma in PC [64,104]. Based on the results for both features, each of the four patterns can be assigned to a different stromal subtype. In FP, the stroma is consistent with the fibrolytic subtype (high αSMA/low collagen), while in TP, PP and CP it is of fibrogenic (low αSMA/high collagen), inert (high αSMA/high collagen), and dormant (low αSMA/low collagen) subtype, respectively (Table 3).

Stromal MMP14 levels were highest in FP and lowest in TP (*p* < 0.01) (Figure 3 and Figure 4). CAV1 was absent in nearly all cases of CP but was expressed at similar levels in the other patterns (*p* < 0.01) (Figure 3 and Figure 4). ITGα5 expression was low or absent in TP and CP but was expressed at high level in PP and FP (*p* < 0.001). Strong expression limited to the immediate peritumoral stroma was observed in PP, whereas expression was diffuse in the stroma of FP (Figure 3 and Figure 4). ITGα2 and ITGβ1 showed a similar differential expression albeit at lower levels compared to ITGα5.

### 2.4. Multiple Patterns within a Single Tumor

Five tumors contained two different patterns—that is, a combination of PP and FP (*n* = 3), PP and TP (*n* = 1), or FP and CP (*n* = 1). One tumor contained three patterns (PP, TP, and CP). The different patterns occurred in separate regions within the tumor mass, each pattern showing the features that define the particular morphology of the cancer cells and stroma. Features for the respective patterns did not differ from those found in tumors that contained only a single pattern.

### 2.5. Prediction of Morphological Pattern

To investigate the robustness of the association between the morphological patterns and the structural and functional features, we tested whether the results for the full set of features would allow prediction of the tumor pattern. Using the *k*-Nearest Neighbors (*k*-NN) algorithm, 97% of the ROIs was assigned to the correct morphological pattern when using a separate holdout dataset containing 30% of the samples for testing. When using cancer-cell features only, classification was correct for 84% of the ROIs, and misclassification occurred for ROIs with any of the four patterns. Prediction of the morphological pattern based exclusively on stromal features resulted in correct classification for 96% of the ROIs, with only 10 of 60 ROIs with a PP morphology being misclassified as either TP or FP. Interestingly, when using only the panel of nine features that are related to the ECM, prediction of the morphological pattern was equally good (97%) as when using the full stromal panel (18 features) or the full panel (26 features) (Table S3).

Feature importance computed by the extremely randomized tree (ERT) algorithm showed that features related to collagen I and collagen III as well as fibronectin were the most important ones for the prediction of the morphological pattern (Figure S2). This is in accordance with the finding that the ECM-related features alone were able to predict the morphological pattern with the same accuracy as the full panel of features. Using ERT gave the same classification accuracy for the holdout dataset as obtained with k-NN (97%).

## 3. Discussion

From the wide spectrum of morphological appearances that exist in PC NOS, four patterns were selected that are common—found in 67% of PC NOS in the current series—and exhibit distinct morphological features of both the cancer-cell and stromal compartments, allowing unequivocal identification on H&E staining. Immuno-/histochemical staining as well as morphometric analysis revealed significant differences between at least two of the four patterns for a panel of 26 features. The latter were investigated in this study given their involvement in structural and functional properties that are deemed important in PC. Furthermore, the features are part of transcriptional signatures that characterize PC subtypes [3,12], with the obvious exception of fiber alignment, epithelial proximity and stromal cell density, as these are topologically defined features.

To the best of our knowledge, this study is the first to report that morphological heterogeneity correlates with structural and functional divergence in tissues of human PC NOS. The findings of this study further demonstrate that the various biological processes, in which the analyzed features are involved, are not uniformly operational in PC and co-vary with the morphological phenotype of the tumor. Indeed, while overall results for the entire study series are in line with observations that are reported in the literature (irrespective of tumor morphology), this study reveals significant divergence between the patterns for each of the analyzed features.

This study is also the first to investigate morphological heterogeneity in the stromal compartment of PC, a phenomenon that has received little attention [9] despite the pivotal role of the stroma in PC [105]. Analysis was limited to the immediate peritumoral stroma rather than the bulk stroma because the former plays a key role in the biology of PC. Moreover, the high prevalence of morphological intratumor heterogeneity also demands that stromal analysis be limited to the juxta-tumoral compartment.

A prominent stroma accounting for up to 80% of the tumor mass is considered a hallmark of PC. However, our study revealed significant differences in stromal content between the four patterns, despite the fact that the study series consisted exclusively of well-differentiated PC with a glandular growth pattern [96,106].

The ECM has a multifarious role by providing structural support as well as biochemical and biophysical cues [107]. While the ECM composition for the overall series was very similar to observations published in the literature [33,63,72,73,82,102,108], our study revealed significant differences between the patterns. In TP, which contained the highest amount of tumor stroma, the ECM was rich in collagens and hyaluronan but lacked fibronectin in nearly all cases. FP and PP, patterns with a moderate amount of stroma, contained comparatively less collagens and hyaluronan but were rich in fibronectin. The very small amount of ECM in CP contained mainly hyaluronan but little collagen and fibronectin. These findings imply that the interstitial pressure, which is mainly determined by the amount of hyaluronan and fibrillary collagen [109,110,111], likely differs between the four patterns. Because hyaluronan is a target of stroma-directed therapy [112,113], the impact of the latter on interstitial pressure and drug delivery may vary considerably between patterns. Recently, fibronectin has been linked to acquired chemoresistance in PC [69]; hence, its high content in FP and PP and absence in TP may be of clinical interest. The stark variation in the amount of collagen between the patterns may explain—at least in part—the conflicting results that have been reported regarding the association between a prominent collagenous stroma and survival [63,64].

Not only the composition but also the spatial organization of the ECM is relevant for its biological function. In particular, alignment of collagen and fibronectin fibers promotes directional cancer cell migration in various cancers, including PC [62,114,115]. While marked fiber alignment has been reported in only 12% of PC [65], we found significant variation between the patterns. In the majority of tumors with TP, the stroma showed marked parallel alignment of collagen fibers, resulting in a taut, tendon-like stromal appearance. In contrast, in FP, the whorled fiber arrangement reflected alignment of fibronectin rather than collagen. Interestingly, ITGα5β1, the receptor for fibronectin which is instrumental in the production, assembly and alignment of fibronectin by the CAFs [115], was highly and diffusely expressed in FP. In PP, fibronectin deposition and ITGα5β1 expression were mainly limited to a thin stromal sheath encircling the cancer glands. In contrast, fibronectin and ITGα5β1 were absent or low in TP and CP. These observations are in accordance with in vitro studies revealing the ITGα5β1-dependence of fibronectin deposition and alignment by CAFs [115,116], but at the same time, our findings indicate that this type of ECM organization is not common to all PC. ITGα2, which together with ITGβ1 forms the main receptor for collagen I [117], was present in all patterns, albeit at different levels. Given the multitude of different integrin heterodimer combinations and their biological roles, further studies are needed to understand the relevance of the observed heterogeneity between patterns. Finally, the density of stromal cells, the vast majority of which were αSMA-positive CAFs, differed significantly, being highest in FP and lowest in CP.

**Figure 5:**
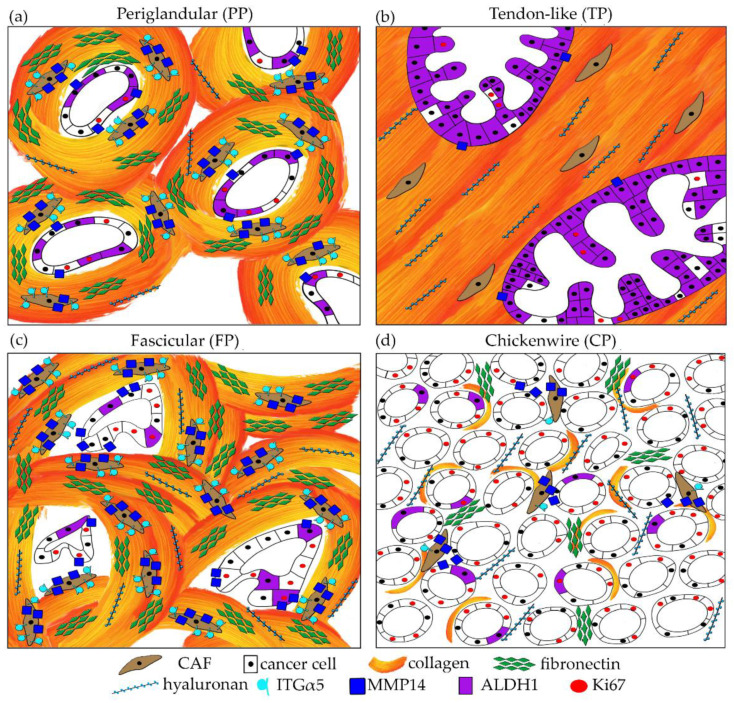
Four patterns of pancreatic cancer with distinctive morphological, structural and functional features of the cancer-cell and stromal compartments. (**a**) In the periglandular pattern, the stromal sheath that encircles the tumor glands contains a moderate amount of CAFs and is rich in fibronectin and MMP14, produced by both CAFs and PCCs. The latter show moderate expression of ALDH1 and Ki67. (**b**) The tendon-like pattern has a collagen- and hyaluronan-rich stroma that is devoid of fibronectin and shows marked fiber alignment. The majority of cancer cells express ALDH1. Note the low levels of MMP14 and Ki67. (**c**) The whorled stroma of the fascicular pattern contains a high number of CAFs with high expression of ITGα5 and marked deposition of aligned fibronectin. Note the high-level expression of MMP14 in stroma and PCCs. (**d**) The small stromal compartment in the chickenwire pattern is relatively rich in hyaluronan but contains little collagen and fibronectin and lacks fiber alignment. CAFs are low in number. Note the high Ki67-expression in the PCCs. ALDH1, aldehyde dehydrogenase 1; CAF, cancer-associated fibroblast; ITGα5, integrin α5; MMP14 matrix metalloproteinase 14; PCC, pancreatic cancer cell.

Emerging from these findings is a picture of distinct structural and functional diversity of the stroma that co-varies with and is to some extent reflected in the morphology of that compartment (Figure 5).

Interestingly, when applying the recently proposed stromal subclassification of PC that is based on the activated stroma ratio, i.e., the ratio between the number of αSMA + CAFs and amount of collagen, each of the four patterns can be assigned to a different stromal subtype [64,104]. Interestingly, the fibrolytic and fibrogenic subtypes observed in FP and TP, respectively, were reported to associate with shorter and longer progression-free survival, respectively, than the inert (PP) and dormant (CP) subtypes [104]. Further results from this study extend the nature of the stromal subtypes: high MMP14-expression by both CAFs and cancer cells in FP fits with a dynamic stroma, undergoing active remodeling. In contrast, low MMP14-levels in TP support the notion of a stable collagen-rich stroma with little remodeling.

The proliferative activity of cancer cells is clinically relevant, as it relates to the growth capacity of the cancer and its possible response to cytotoxic treatment. While the median value of the Ki67-index in the overall series was near-identical with that reported by others [97,98], the four patterns differed significantly between themselves. It was highest in CP, which would indicate a more aggressive nature of tumors with CP morphology, despite the preserved glandular growth pattern that defines CP as well differentiated [96]. The lack of co-variance between grade of differentiation and proliferative activity in PC was recently highlighted [106]. The high expression level of CAV1 in the cancer cells of CP compared to the other patterns, points also at a more aggressive behavior, as CAV1-expression has been associated with cancer cell proliferation and migration, chemoresistance and worse patient outcome [25,26,27]. The mutually exclusive CAV1-expression in cancer cells and stroma observed in this study has been described previously but remains as yet unexplained [24].

As in other cancers, cancer stem cells in PC drive metastasis and recurrence, the latter by endowing resistance to chemo(radio-)therapy. While expression of the three CSC markers in the overall series was similar to observations by others [13,17,18,30,34,35,39,40,50,51,99], results differed significantly between patterns. The most prominent difference was observed for ALDH1, which was strongly expressed in nearly all TP, but absent or expressed at low level in the other patterns. Furthermore, expression significantly lower than that reported in the literature was observed for CD44 (in PP) and CD133 (in TP and CP). Interestingly, previous reports commented on heterogeneity of CSC marker expression in association with morphological tumor heterogeneity [34,50].

Taken together, the presence of features that are linked to key tumor biological processes varies between the cancer cell compartments of the four patterns. In CP, high proliferative activity and high-level expression of CD44 and CAV1 suggest a more aggressive tumor behavior. While FP shows a lower proliferation than CP, its cancer cells express high levels of two CSC markers (CD44, CD133) as well as MMP14. The latter may have pro-oncogenic effects outside its well-documented role in cell motility and invasion, namely increased metabolism and proliferation [118]. TP is characterized by high-level expression of ALDH1, which is associated with worse patient outcome and resistance to chemo(radio-)therapy. PP shows low proliferation and low-level expression of CSC markers, CAV1 and MMP14.

The analysis shows that the four morphological patterns differ significantly and consistently in most of the features that were analyzed. Conversely, our findings demonstrate that the features form a robust signature that allows correct prediction of the morphological pattern in 97% of ROIs.

In six of the 39 (15%) cases included in the study, two or three of the four patterns were present within the same tumor. The signature of stromal and cancer-cell features of a pattern that co-occurred with other patterns did not differ from that observed in tumors containing only one pattern. Moreover, most cases in this series contained to a varying extent one or several morphological phenotypes other than the four that were investigated. These observations illustrate that morphological intratumor heterogeneity is common, as reported by others [9,10,119]. Collectively, observations in this study concur with the widespread intratumor heterogeneity that has been described recently at the transcriptional and proteomics level [120,121,122]. Moreover, intratumor heterogeneity has also been reported for several of the individual features analyzed in this study: amount of hyaluronan [77,102] and collagen [19], degree of collagen alignment [19,65], expression of CD44 [34], CD133 [50], ALDH1 [99] and MMP14 [123], and proliferative activity [97,106]. While in these studies intratumor heterogeneity for individual features was reported without further characterization of the morphological phenotype of PC, the results of our study indicate that features do not vary randomly between and within tumors but rather co-vary with the tumor morphology.

Phenotypic heterogeneity in cancer is a complex multifactorial phenomenon that results from the integration of genetic, epigenetic and environmental inputs [124,125]. As such, the histomorphology of cancer, including PC, contains rich, highly integrated information that, in contrast to multiomics data, also visualizes the topology of the various tumor features. Deciphering the complex information contained in histomorphology in terms of discrete biological processes at the molecular level has only just started, to some extent prompted by the application of artificial intelligence [126]. Obviously, the association between morphology, tumor behavior and patient outcome is complex and multifactorial, with a possible divergent impact from the cancerous and stromal compartments.

The results of this study have potentially important implications. First, the study reveals significant and possibly clinically relevant differences between morphological phenotypes that currently remain undistinguished as they are lumped together in the large group of PC NOS. While the four patterns analyzed in this study were selected to test the hypothesis that morphology and functional aspects are linked, the development of a morphological classification for PC would require the characterization of a larger number of morphological phenotypes in PC over and above those currently considered by the WHO classification. The prevalence and complexity of morphological heterogeneity in PC is supported by recent studies that reveal marked intratumor heterogeneity at the transcriptional level, implying that current classification systems may offer a too simplistic taxonomy of PC [120,121,127]. By the same token, intratumor heterogeneity hampers the study of the prognostic significance of the various morphological phenotypes.

Second, the findings of this study question the widespread use of a single “representative” tumor block for bulk molecular analysis. The need for higher cancer cell density in order to avoid contamination with nonneoplastic cells may lead to the selection of tumor areas with low stromal volume, such as CP, and result in overrepresentation of biological mechanisms that are active in such phenotypes.

Third, while molecular assessment of intratumor heterogeneity remains challenging, pathology examination allows expedient and low-cost assessment of the extent and nature of morphological heterogeneity within a tumor. Especially the evaluation of heterogeneity in morphological features that are relevant regarding the effect of targeted treatment (e.g., hyaluronan content for hyaluronidase therapy [112,113]) may be of clinical interest.

Fourth, this study reveals that the tumor stroma, which hitherto is ignored in pathology diagnostics for PC, exhibits distinctive morphological features that may inform on relevant underlying processes. Given that accumulating evidence supports a link between the biological properties of the peritumoral stroma and clinical aspects of PC [6,101,105], patient stratification based also on stromal morphology could be clinically more relevant.

Recent studies have demonstrated a mutual shaping of the cancer cell and stromal compartments. While the cancer cell genotype tunes the composition and biophysical properties of the tumor stroma [100], the ECM and CAFs exert epigenetic effects on the cancer cells and shape the tumor architecture [101,107]. Recent transcriptomics-based studies present evidence for a close link between the phenotype of the stroma and the cancer cells [12,128] rather than an independent combination of both [3]. The results of this study show that the morphological phenotype of PC in the four patterns is determined by a particular combination of both stromal and cancer cell-related features rather than either in isolation and independently of each other. Indeed, using exclusively the set of stromal features resulted in a similarly good prediction of the tumor pattern, that is, the morphological phenotype including both cancer cells and stroma.

Our study has a number of limitations. From the wide range of morphological phenotypes, only four were analyzed. As the four selected patterns represent low-grade PC, our observations may not be pertinent to high-grade, that is, poorly differentiated tumors. The analysis was based on a limited set of features that did not represent important processes such as epithelial-mesenchymal transition or immune response. The number of biochemical and biophysical properties that are highlighted by in vitro and in vivo studies as potentially clinically relevant is large and requires extensive analysis beyond this initial proof-of-concept study. Last but not least, investigations at genomic and/or transcriptional level were not included in this study, but 14 of the non-topological features are part of transcription-based signatures that are used for molecular classification of PC [3,12]. Future studies correlating genomics and transcriptomics data with a variety of morphological patterns are needed to understand the molecular basis of morphological heterogeneity.

## 4. Materials and Methods

### 4.1. Tissues

Archival H&E-stained histology slides from consecutive cases of treatment-naïve PC resected between December 2017 and April 2019 at Oslo University Hospital, 0424 Oslo, Norway, were reviewed. For each case, tissue blocks containing one of the four patterns were selected. Areas with necrosis or inflammatory cell infiltration were excluded, as this affects the composition of the stromal component [12,104].

### 4.2. Cancer Cell and Stromal Features

The cancer cells were analyzed for proliferative activity (Ki67) and expression of the CSC markers CD44, CD133, and ALDH1. The stromal compartment was investigated for the total amount of collagen, collagen fiber alignment, amount and deposition pattern of collagen I and III, fibronectin and hyaluronan; density of CAFs, expression of αSMA, and epithelial proximity, the latter as a measure for stromal volume [129]. Cancer-stroma interactions were investigated for expression of ITGα2, α5, and β1. MMP14 and CAV1 were assessed in both compartments.

### 4.3. Histochemistry and Immunohistochemistry

Histochemical and immunohistochemical staining of formalin-fixed paraffin-embedded, 3.5 µm thick, serially cut, whole-tissue sections was done manually using standard protocols as previously described [130]. Briefly, tissue sections were incubated with primary antibodies at 4 °C overnight, followed by incubation with secondary antibodies as outlined in Table 4. Endogenous peroxidase activity and unspecific antibody binding were blocked with EnVision Flex peroxidase blocking agent (Dako, Glostrup, Denmark, catalogue nr. DM841) and 1% BSA, respectively.

Histochemistry using hyaluronic acid binding protein was employed for the histochemical detection of hyaluronan, as previously described (see Table 4) [82]. Fibrillar collagen was detected with ready-to-use picrosirius red staining. Hematoxylin was used for counterstaining.

Appropriate positive and negative controls were used to verify specificity of all tested markers. Negative control samples were incubated with PBS instead of the primary antibody.

### 4.4. Semiquantitative Scoring

Sections were viewed with a light microscope (Nikon eclipse Ni, Amsterdam, Netherlands) using polarized light for visualization of collagen fibers with picrosirius red staining [103,131]. In each tumor, at least five randomly selected ROIs representative of a particular pattern were photographed at 200x magnification (camera: Infinity2–5C Lumenera, Ottawa, ON, Canada). Picture acquisition was done with strict compliance regarding exposure time, light intensity, contrast, aperture, and sensor sensitivity.

From the wealth of different systems and threshold values that have been used previously to score histochemical (hyaluronan) and immunohistochemical staining for any of the markers included in the panel of this study, the following uniform, simple and robust staining score was selected: 0 = no staining, 1 = staining in <25% of the relevant compartment, 2 = staining in 26–50% of the relevant compartment, 3 = staining in >50% of the relevant compartment. Staining scores 0 and 1 were regarded as “low,” score 2 as “medium,” and score 3 as “high,” as previously described [24,82,132,133,134]. Because of major variation in both staining intensity and number of positive cells, immunostaining for CSC markers was scored by multiplying intensity (0 = negative, 1 = low, 2 = moderate, 3 = high) and extent of staining (0 = 0%, 1 = 1–10%, 2 = 11–50%, 3 = 51–80%, 4 ≥ 81%), resulting in an immunoreactive score (IRS) between 0 and 12 [34,36,39,135]. The IRS was categorized as negative (0), low (1–5), and high (6–12). The pattern of deposition of ECM-components was classified as diffuse or peritumoral, the latter denoting deposition immediately surrounding the tumor glands.

### 4.5. Quantitative Assessment

The total amount of fibrillar collagen and the alignment of collagen fibers were quantified with ImageJ (NIH, Bethesda, MD, USA) and the OrientationJ plugin [136], respectively, using the polarization photographs of picrosirius red-stained sections that were converted to grey-scale 8-bit images. Identical analysis settings and thresholds were used for all cases. Higher values corresponded with higher fibrillar collagen content (reflecting the area fraction of polarized light) and better fiber alignment, respectively. Stromal cells showing features characteristic of fibroblasts, that is, a spindle cell shape and an elongated nucleus without atypical features, were counted on H&E-sections. The average was calculated from cell counts in five random 0.01 mm^2^ rectangular areas per ROI, excluding foci with inflammatory cell infiltration. Epithelial proximity is a feature that denotes the extent of contiguous areas of stroma that separate cancer cell clusters and has been used previously for the morphological analysis of the stroma in cancer [129]. It was assessed with ImageJ based on the average of shortest distances between any two cancer glands present in an ROI. Immunostaining for Ki67 was counted in the cancer cell population and expressed as the percentage of positive cells.

### 4.6. Network Visualization

Network visualization of the results for all features was generated with Gephi (v0.9.2) using the Fruchterman–Reingold algorithm [137] and an adjacency matrix (RStudio v 1.453). The former is a force-based approach using small to medium-sized networks (unweighted, undirected graphs) [138,139]. The vertex layout is determined by attractive and repulsive “forces” between the vertices.

### 4.7. Statistical Analysis

The Kruskal–Wallis multiple comparison test and the Mann–Whitney U test for pair-wise comparison were used to test whether the feature data differed significantly between the four morphological patterns. These are nonparametric methods for analyzing whether two or more datasets come from the same statistical distribution or from different distributions. In this study, *p*-values < 0.05 were considered significant for detection of a difference. The tests were carried out using the SPSS^®^ software for Windows (v22) (IBM SPSS Statistics, Armonk, NY, USA).

### 4.8. Prediction of Morphological Pattern

In order to test the strength of the association between morphology and the panel of features, we investigated whether the morphological pattern in each ROI could be predicted based on the features used in this study. The samples were classified with respect to the four different morphological patterns using the *k*-nearest neighbors algorithm (*k*-NN) [140], with *k* equal to 3. According to this algorithm, a sample is classified by a majority vote of its *k* neighbors, with the object being assigned to the class most common among its *k* nearest neighbors, and each neighbor is attributed a weight of 1/*d*, where *d* is the distance to the neighbor. The classification was done with four different sets of features: (i) all features, (ii) only cancer-cell features, (iii) only stromal features and iv) only ECM-related features. Each classification model was trained using 70% of the samples, while a separate holdout set of 30% of the samples (randomly selected) was used to test the prediction accuracy.

In order to further investigate the importance of the various features for predicting the morphological pattern, the extremely randomized tree (ERT) algorithm for estimation of the feature importance was applied [141]. The ERT algorithm is a tree-based ensemble classification method by which the outcomes from many decision trees are averaged to obtain a final output. Decision trees generate decision rules to divide observations into segments that have the largest difference with respect to the target variable. Each rule selects both the variable and the best breakpoint to separate the resulting subgroups maximally, and one seeks to find the smallest set of rules that is consistent with the training data. The ExtraTreesClassifier implementation in the SCiKit-learn module “ensemble”, where Python v. 3.7.4. was used [142,143].

## 5. Conclusions

This proof-of-concept study is the first to show that heterogeneity in morphological features reflects structural and functional heterogeneity and that tumors exhibiting the same histological pattern also share functional and structural properties. Furthermore, this study demonstrates that biological processes deemed to be crucial in PC may not be relevant in all PC tumors nor in all parts of a tumor, considering that morphological inter- and intratumor heterogeneity is common. Further systematic investigation of morphological heterogeneity and its link with biological diversity, treatment effect and patient outcome may ultimately allow pathologists to provide new and clinically meaningful information on tumor-intrinsic properties as part of the routine diagnostic work-up.

## Figures and Tables

**Table 1:** Experimental observations and clinical relevance of the listed features in pancreatic cancer.

Feature	Experimental Observations	Clinical Relevance
ALDH1	Mediates resistance against chemo(radio-)therapy [13,14,15] Enrichment of ALDH1+ PCCs following gemcitabine treatment [16]	Correlates with poor survival [15,17] Low expression correlates with poor survival [18]
αSMA	Correlation with aligned collagen [19]	Correlates with survival [20,21]
CAV1	Modulates response to substrate stiffness and stromal remodeling [22,23] Promotes PCC proliferation, invasion, treatment resistance [24,25]	High expression in PCCs correlates with larger tumor size, higher differentiation grade, worse outcome [25,26] Loss of stromal expression correlates with stage [27]
CD44	Confers CSC phenotype Interaction with HA promotes PCC migration, MMP14-upregulation [28], resistance to gemcitabine [29,30], metastatic potential [31,32]	Correlates with early recurrence, poor survival, advanced stage, lower grade [30,33,34,35,36,37,38]; no correlation with survival, advanced stage, differentiation grade [39,40] Increase of CD44 + PCCs after gemcitabine treatment [41,42]
CD133	Induces stemness [43], EMT [44,45,46], altered metabolic profile and chemoresistance [44,47]	Correlates with metastasis and survival [40,48,49] No correlation with survival, stage, differentiation grade [39,40,50] Numerous CD133+ PCCs after gemcitabine treatment correlates with poor outcome [42,51]
Col I	Promotes proliferation, migration and metastasis [52,53,54,55,56,57,58,59] Reduces sensitivity to gemcitabine and 5-FU [54,58] Alignment enhances migration, invasive velocity and directionality [60,61,62]	High content correlates with poor survival [63], with longer survival [64] High alignment correlates with poorer survival independent of traditional prognostic determinants [65]
Col III	Promotes proliferation and migration [66]	Pretreatment serum levels correlate with survival [67,68]
FN	Affects resistance to chemotherapy [69]	Correlates with higher stage, poorer survival [70,71] No correlation with survival [72,73]
HA	Increases interstitial pressure and influences drug delivery [60,74,75,76,77] Promotes PCC proliferation, motility and metastasis via binding to CD44 [78,79,80,81]	Correlates with rapid tumor progression [82] and survival [63,83]; no correlation with survival [33,82] Treatment with recombinant hyaluronidase and gemcitabine prolongs survival in patients with HA-rich tumors [84]
ITGα2β1	Mediates collagen I-induced proliferation and migration of PCCs [59,85] ITGβ1-blocking inhibits invasiveness of PCCs [62]	Overexpression correlates with poorer overall and disease-free survival, higher grade and stage [86]
ITGα5β1	Mediates PCC migration along fibronectin and increased PCC survival [87,88]	Overexpression of ITGα5 correlates with poorer overall survival [21]
MMP14	Enables PCC migration and release of growth factors, incl. TGFβ [89,90] Sheds cell-surface biomolecules, incl. CD44 [91] Upregulation sensitizes to gemcitabine [92,93]	Enhanced expression in metastasis compared to primary PC [94,95]

ALDH1, aldehyde dehydrogenase 1; αSMA, α-smooth muscle actin; CAV1, caveolin-1; Col, collagen; CSC, cancer stem cell; EMT, epithelial-mesenchymal transition; FN, fibronectin; HA, hyaluronan; ITG, integrin; MMP14, matrix metalloproteinase 14; PCC, pancreatic cancer cell; TGFβ, transforming growth factor β; 5FU, fluorouracil.

**Table 2:** Statistical results for the panel of features compared between the tumor patterns. Low *p*-values (<0.05) indicate significant differences between the feature data corresponding to the different morphological patterns.

Feature	Compart-ment	Kruskal-Wallis Test	Mann–Whitney U Test					
			PP vs. TP	PP vs. FP	PP vs. CP	TP vs. FP	TP vs. CP	FP vs. CP
ALDH1	C	<0.001	<0.001	NS	NS	<0.001	<0.001	NS
αSMA	S	<0.001	<0.001	0.01	<0.001	<0.001	0.02	<0.001
CAV1	C	<0.001	NS	NS	<0.001	NS	<0.001	<0.001
CAV1	S	<0.001	<0.001	0.006	<0.001	NS	<0.001	<0.001
CD133	C	<0.001	<0.001	NS	<0.001	<0.001	NS	<0.001
CD44	C	0.004	0.003	0.007	0.001	NS	NS	NS
Col align	S	0.001	0.001	0.011	0.001	<0.001	<0.001	NS
Col I	S	<0.001	0.033	<0.001	<0.001	<0.001	<0.001	<0.001
Col I pattern	S	<0.001	<0.001	<0.001	<0.001	NS	<0.001	<0.001
Col III	S	<0.001	<0.001	<0.001	<0.001	<0.001	<0.001	<0.001
Col III pattern	S	<0.001	<0.001	<0.001	<0.001	NS	<0.001	<0.001
Ep prox prox	S	<0.001	<0.001	<0.001	<0.001	<0.001	<0.001	<0.001
FN	S	<0.001	<0.001	0.02	<0.001	<0.001	<0.001	<0.001
FN pattern	S	<0.001	<0.001	<0.001	0.009	<0.001	<0.001	<0.001
HA	S	<0.001	<0.001	0.002	<0.001	<0.001	NS	<0.001
ITGα2	C	<0.001	<0.001	0.001	<0.001	NS	NS	NS
ITGα2	S	<0.001	0.002	NS	<0.001	0.022	NS	0.002
ITGα5	S	<0.001	<0.001	NS	<0.001	<0.001	<0.001	<0.001
ITGα5 pattern	S	<0.001	<0.001	<0.001	<0.001	<0.001	<0.001	<0.001
ITGβ1	C	<0.001	<0.001	<0.001	<0.001	NS	<0.001	<0.001
ITGβ1	S	<0.001	<0.001	NS	<0.001	<0.001	NS	<0.001
Ki67	C	<0.001	<0.001	<0.001	<0.001	<0.001	<0.001	<0.001
MMP14	C	<0.001	0.001	<0.001	0.037	<0.001	<0.001	<0.001
MMP14	S	<0.001	<0.001	<0.001	NS	<0.001	<0.001	<0.001
Stromal cell density	S	<0.001	<0.001	0.008	<0.001	<0.001	<0.001	<0.001
Total collagen	S	<0.001	<0.001	<0.001	<0.001	<0.001	<0.001	NS

ALDH1, aldehyde dehydrogenase 1; αSMA, α-smooth muscle actin; C, Cancer; CAV1, caveolin-1; Col, collagen; Col align, collagen alignment; CP, chicken-wire pattern; Ep prox, epithelial proximity; FN, fibronectin; FP, fascicular pattern; HA, hyaluronan; ITG, integrin; MMP14, matrix metalloproteinase 14; NS, not significant (*p* ≥ 0.05); PP, periglandular pattern; S, stroma; TP, tendon-like pattern.

**Table 3:** Activated stroma index in the four tumor patterns. The index is the ratio between αSMA expression (immunohistochemically assessed) and the total amount of collagen (morphometric evaluation), averaged for each pattern. Assignment to the stromal subtypes is according to [104]. Kruskal-Wallis testing showed significant difference in activated stroma index between the four patterns (*p* < 0.01). αSMA, α-smooth muscle actin.

Tumor Pattern	αSMA (Staining Score)	Total Collagen (Area Fraction %)	Activated Stroma Index	Stromal Subtype
Periglandular	High (2.60)	High (16.95)	0.27	Inert
Tendon-like	Low (1.50)	High (33.21)	0.05	Fibrogenic
Fascicular	High (2.90)	Low (7.35)	1.35	Fibrolytic
Chickenwire	Low (1.20)	Low (3.60)	0.56	Dormant

**Table 4:** Antibodies and procedures for immunohistochemical and histochemical staining.

Immunohistochemical Staining							
Primary Antibodies							
Antigen	Company	Cat #	Antigen Retrieval	Dilution	Incubation	Species	Control Tissue
αSMA	Abcam	ab5694	CB, 100 °C	1:100	ON, 4 °C	Rabbit	Blood vessel
ALDH1A1	Abcam	ab52492	CB, 100 °C	1:800	ON, 4 °C	Rabbit	Kidney, lung
Caveolin-1	Abcam	ab32577	CB, 100 °C	1:750	ON, 4 °C	Rabbit	Liver, lung
CD133/1	Miltenyi Biotec	130–108-062	CB, 100 °C	1:100	ON, 4 °C	Mouse	Gallbladder
CD44	Dako	M7082	CB, 100 °C	1:200	ON, 4 °C	Mouse	Gallbladder
Col Iα1	LifeSpan BioSciences	LS-B5932	CB, 100 °C	1:200	ON, 4 °C	Mouse	Prostate
Col III	Abcam	ab7778	CB, 100 °C	1:300	ON, 4 °C	Rabbit	Testis
Fibronectin	Abcam	ab6328	TEB, 100 °C	1:300	ON, 4 °C	Mouse	Granulation tissue
ITGα2	Abcam	ab133557	CB, 100 °C	1:1000	ON, 4 °C	Rabbit	Testis
ITGα5	Abcam	ab150361	CB, 100 °C	1:200	ON, 4 °C	Rabbit	Placenta
ITGβ1	ThermoFisher	MA5-17103	CB, 100 °C	1:200	ON, 4 °C	Mouse	Small intestine
Ki67	Dako	M7240	CB, 100 °C	1:1000	ON, 4 °C	Mouse	Small intestine
MMP14	Abcam	ab51074	TEB, 100 °C	1:1600	ON, 4 °C	Rabbit	Placenta
**Secondary Antibodies**							
	**Company**	**Cat #**	**Antigen Retrieval**	**Dilution**	**Incubation**	**Detection**	**Control Tissue**
Immpress HRP Anti-Rabbit IgG	Vector Labs	MP-7401	-	Ready to use	1 h, RT	DAB Peroxidase substrate kit (SK-4100)	-
Immpress HRP Anti-Mouse IgG	Vector Labs	MP-7402	-	Ready to use	1 h, RT	DAB Peroxidase substrate kit (SK-4100)	-
**Histochemical Staining**							
Hyaluronan	Merck Millipore	385911	CB, 60 °C	1:100	1 h, RT	ABC-AP-Kit (Vector Labs, AK-500) and Liquid Permanent Red (Dako, K0640)	Cartilage
Picrosirius red	Histolab	HL27150.0500	-	Ready to use	10 min, RT		Tendon

CB, Citrate buffer (pH 6); TEB, Tris/EDTA buffer (pH 9). Abcam, Cambridge, UK; Histolab, Askim, Sweden; LifeSpan Biosciences, Seattle, WA, USA; Merck Millipore, Burlington, MA, USA; Miltenyi Biotec, Bergisch Gladbach, Germany; Thermo Fisher Scientific, Waltham, MA, USA; Vector Labs, Burlingame, CA, USA; Dako: Agilent Technologies, Glostrup, Denmark; ThermoFisher: Waltham, MA USA.

## Data Availability

The data presented in this study are available on request from the corresponding author. The data are not publicly available due to ethical considerations and data regulations.

## Supplementary Materials

The following are available online at https://www.mdpi.com/2072-6694/13/4/895/s1, Figure S1: Histological features of the four morphological patterns, Figure S2: Feature importance estimation based on the ERT algorithm, Table S1: Clinicopathological features of the study series of resected, treatment-naïve human pancreatic cancer (*n* = 39), Table S2: Results for the panel of features for the series overall and for each tumor pattern individually, Table S3: Correct and incorrect prediction of morphological pattern based on the full set or on subsets of the analyzed features.

## Abbreviations

ALDH1	Aldehyde dehydrogenase 1
αSMA	α-Smooth muscle actin
CAF	Cancer-associated fibroblast
CAV1	Caveolin-1
Col	Collagen
CP	Chickenwire pattern
CSC	Cancer stem cell
ECM	Extracellular matrix
EMT	Epithelial-mesenchymal transition
ERT	Extremely randomized tree
FN	Fibronectin
FP	Fascicular pattern
HA	Hyaluronan
H&E	Hematoxylin and eosin
IRS	Immunoreactive score
ITG	Integrin
MMP14	Matrix metalloproteinase 14
NOS	Not otherwise specified
PC	Pancreatic cancer
PCC	Pancreatic cancer cell
PP	Periglandular pattern
ROI	Region of interest
SS	Staining score
TP	Tendon-like pattern
