# Supplementary material for: Morphological Heterogeneity in Pancreatic Cancer Reflects Structural and Functional Divergence
Source: Cancers (Basel). 2021 Feb 20;13(4):895. doi: 10.3390/cancers13040895 (PMC7924365; doi:10.3390/cancers13040895)
Supplement: Supplementary file 1 [file cancers-13-00895-s001.pdf]

cancers

Supplementary Material: Morphological Heterogeneity in Pancreatic Cancer Reflects Structural and Functional Divergence

Petra Sántha, Daniela Lenggenhager, Anette Finstadsveen, Linda Dorg, Kristin Tøndel, Manoj Amrutkar, Ivar P. Gladhaug, and Caroline Verbeke

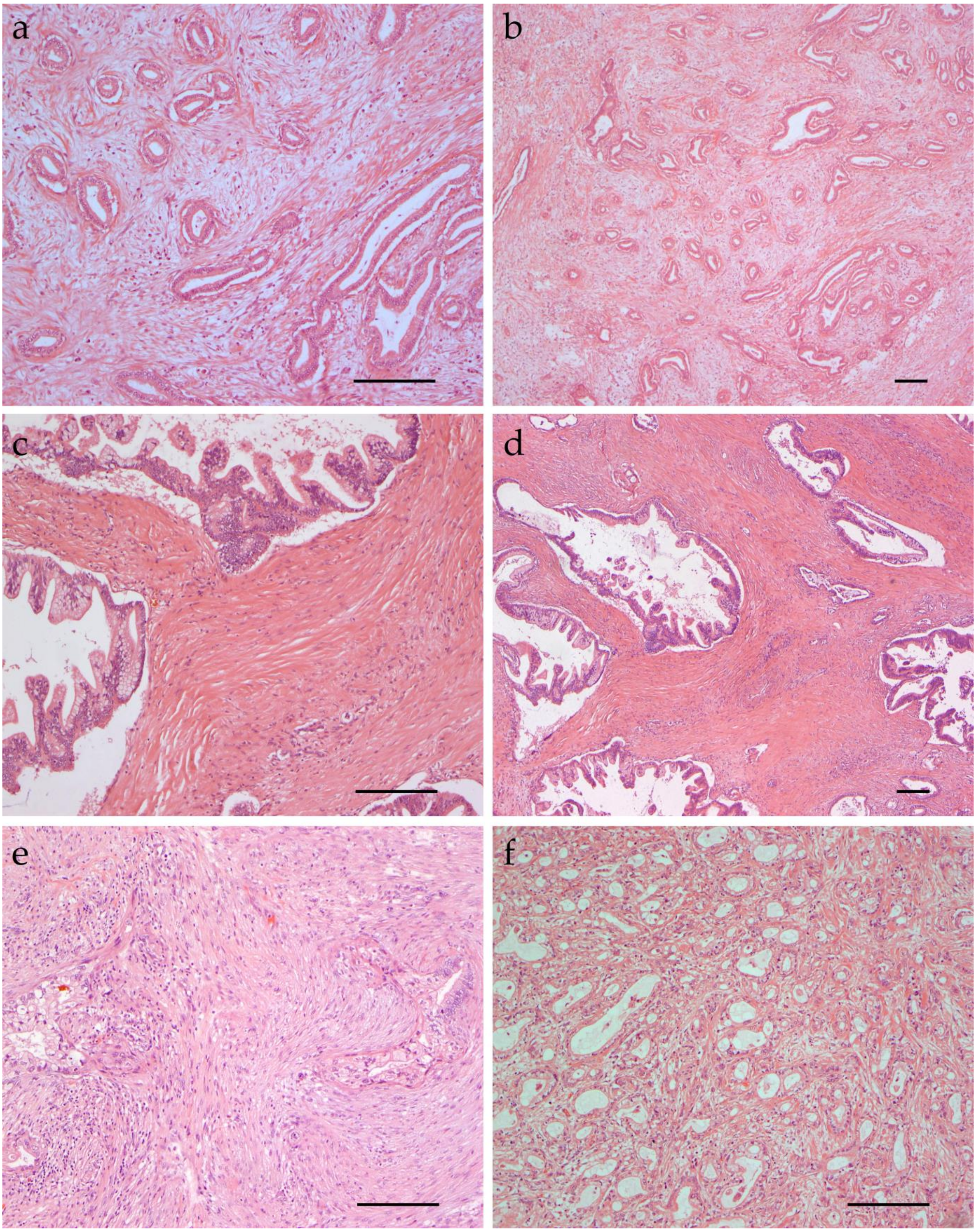

Figure S1. Histological features of the four morphological patterns. (**a**) The periglandular pattern consists of simple glands with little if any branching. (**b**) The stroma is moderately cellular and shows a thin rim of a more condensed, eosinophilic stroma that surrounds the individual glands. On lower magnification, it is readily appreciated that the tumor glands are medium-sized and separated by a moderate amount of stroma. (**c**) In the tendon-like pattern, the tumor glands are large, and the cancer cells form tufts and micropapillary projections into the glandular lumen, resulting in a cystopapillary appearance. The surrounding stroma is reminiscent of tendon tissue, as it is dense and cell-poor but deeply eosinophilic and rich in collagen fibers that are fully aligned. (**d**) At lower power, the large size of the tumor glands and the wide stretches of intervening stroma can be appreciated. (**e**) In the fascicular pattern, the tumor glands are relatively small and often angulated, and they are more difficult to discern from the surrounding stroma. The latter is highly cellular and shows a prominently whorled arrangement, while its more basophilic tinge indicates a relatively low collagen content. (**f**) The chicken-wire pattern is characterized by very small tumor glands that are densely packed and surrounded by a minimal amount of stroma. (H&E staining, scale bar = 200 µm.)

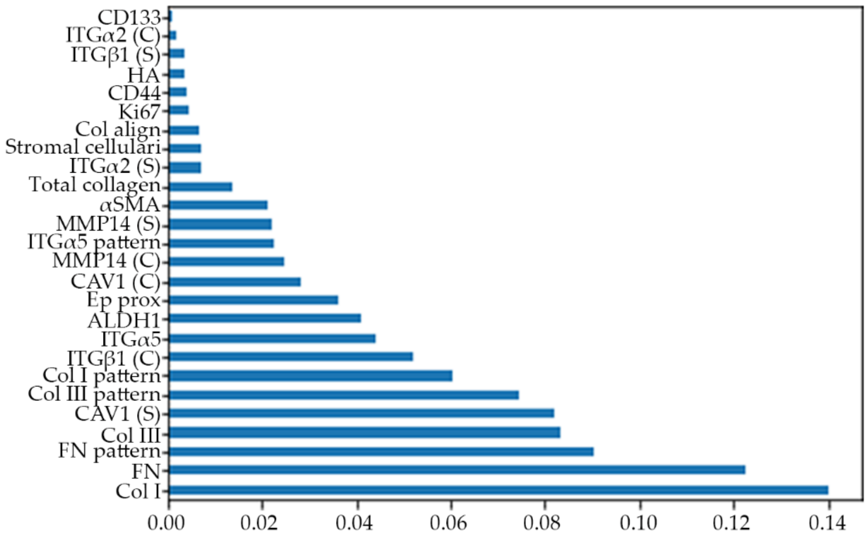

Figure S2. Feature importance estimation based on the extremely randomized tree (ERT) algorithm. ALDH1, aldehyde dehydrogenase 1; αSMA, α-smooth muscle actin; C, Cancer; CAV1, caveolin-1; Col, collagen; Col align, collagen alignment; Ep prox, epithelial proximity; FN, fibronectin; FP, fascicular pattern; HA, hyaluronan; ITG, integrin; MMP14, matrix metalloproteinase 14; Stromal cellular, stromal cellularity.

Table S1. Clinicopathological features of the study series of resected, treatment-naïve human pancreatic cancer (*n* = 39).

**Clinicopathological Features**	
Age (years) median (range)	68 (45–85)
Sex (male/female)	17/22
Tumor site:	
• head of pancreas (%)	32 (82)
• body of pancreas (%)	3 (8)
• tail of pancreas (%)	4 (10)
Tumor size (mm) median (range)	36 (21–55)
pT-stage*:	
• T1 (%)	0
• T2 (%)	27 (69)
• T3 (%)	12 (31)
• T4 (%)	0
pN-stage*:	
• N0 (%)	4 (10)

• N1 (%)	12 (31)
• N2 (%)	23 (59)
Lymphatic invasion:	
• no (%)	3 (8)
• yes (%)	36 (92)
Vascular invasion:	
• no (%)	16 (41)
• yes (%)	23 (59)
Perineural invasion:	
• no (%)	3 (8)
• yes (%)	36 (92)
Margin status:	
• no (%)	9 (23)
• yes, microscopic (%)	29 (77)
• yes, macroscopic (%)	0

* According to TNM UICC 8th. edition [142].

Table S2. Results for the panel of features for the series overall and for each tumor pattern individually.

	**Overall Series (*n* = 233)**	**PP (*n* = 68)**	**TP (*n* = 60)**	**FP (*n* = 50)**	**CP (*n* = 55)**
ALDH1 (IRS):					
average (SD)	3.1 (4.0)	1.2 (1.4)	9 (3)	1.1 (1.3)	0.8 (1.5)
median (range)	2 (0–12)	0 (0–4)	9 (4–12)	0 (0–4)	0 (0–6)
negative	101 (43%)	35 (51%)	0 (0%)	27 (54%)	39 (71%)
low	79 (34%)	33 (49%)	8 (13%)	23 (46%)	25 (27%)
high	53 (23%)	0 (0%)	52 (87%)	0 (0%)	1 (2%)
αSMA (SS):					
average (SD)	2.0 (0.9)	2.6 (0.7)	1.5 (0.8)	2.9 (0.3)	1.2 (0.4)
median (range)	2 (1-3)	3 (1-3)	1 (1-3)	3 (2-3)	1 (1-2)
low	92 (39%)	8 (12%)	39 (65%)	0 (0%)	45 (82%)
medium	41 (18%)	13 (19%)	12 (20%)	6 (12%)	10 (18%)
high	100 (43%)	47 (69%)	9 (15%)	44 (88%)	0 (0%)
Caveolin-1 (SS):					
(C) intensity					
average (SD)	0.6 (1.1)	0 (0)	0 (0)	0 (0)	2.4 (1)
median (range)	0 (0–3)	0 (0–0)	0 (0–0)	0 (0–0)	3 (0–3)
low	183 (79%)	68 (100%)	60 (100%)	50 (100%)	5 (9%)
medium	5 (2%)	0 (0%)	0 (0%)	0 (0%)	5 (9%)
high	45 (19%)	0 (0%)	0 (0%)	0 (0%)	45 (82%)
(S) intensity					
average (SD)	1.9 (1.2)	1.9 (0.7)	2.7 (0.6)	2.6 (0.5)	0.1 (0.3)
median (range)	2 (0–3)	2 (1–3)	3 (1–3)	3 (2–3)	0 (0–1)
low	51 (22%)	0 (0%)	0 (0%)	0 (0%)	51 (93%)
medium	22 (9%)	15 (22%)	3 (5%)	0 (0%)	4 (7%)
high	160 (69%)	53 (78%)	57 (95%)	50 (100%)	0 (0%)
CD133 (IRS):					
Average (SD)	2.2 (3.2)	3.2 (3.2)	1.1 (2.6)	4.1 (3.6)	0.4 (0.9)
median (range)	0 (0–12)	2 (0–9)	0 (0–12)	3.5 (0–12)	0 (0–4)

negative	123 (53%)	22 (32%)	44 (73%)	12 (24%)	45 (82%)
low	59 (25%)	22 (32%)	12 (20%)	15 (30%)	10 (18%)
high	51 (22%)	24 (36%)	4 (7%)	23 (46%)	0 (0%)
CD44 (IRS):					
average (SD)	4.3 (3.7)	2.8 (2.9)	4.2 (2.7)	5.0 (4.0)	5.8 (4.4)
median (range)	4 (0–12)	2 (0–9)	4 (0–9)	6 (0–12)	6 (0–12)
negative	40 (17%)	17 (25%)	6 (10%)	10 (20%)	7 (13%)
low	94 (40%)	34 (50%)	30 (50%)	14 (28%)	16 (29%)
high	99 (43%)	17 (25%)	24 (40%)	26 (52%)	32 (58%)
Col I (SS): intensity					
average (SD)	2.4 (0.9)	2.9 (0.3)	3.0 (0)	2.4 (0.5)	1.0 (0)
median (range)	3 (1–3)	3 (1–3)	3 (3–3)	2 (2–3)	1 (1–1)
low	56 (24%)	1 (1%)	0 (0%)	0 (0%)	55 (100%)
medium	36 (15%)	4 (6%)	0 (0%)	32 (64%)	0 (0%)
high	141 (61%)	63 (93%)	60 (100%)	18 (36%)	0 (0%)
pattern					
peritumoral	85 (36%)	30 (44%)	0 (0%)	0 (0%)	55 (100%)
diffuse	148 (64%)	38 (56%)	60 (100%)	50 (100%)	0 (0%)
Col III (SS): intensity					
average (SD)	1.8 (0.7)	1.9 (0.2)	2.67 (0.48)	1.46 (0.50)	1.0 (0)
median (range)	2 (1–3)	2 (1–2)	3 (2–3)	1 (1–2)	1 (1–1)
low	84 (36%)	2 (3%)	0 (0%)	27 (54%)	55 (100%)
medium	109 (47%)	66 (97%)	20 (33%)	23 (46%)	0 (0%)
high	40 (17%)	0 (0%)	40 (67%)	0 (0%)	0 (0%)
pattern					
peritumoral	101 (43%)	46 (68%)	0 (0%)	0 (0%)	55 (100%)
diffuse	132 (57%)	22 (32%)	60 (100%)	50 (100%)	0 (0%)
Collagen fiber alignment:					
average (SD)	25 (22)	28 (24)	44 (22)	14 (12)	10 (8)
median (range)	16 (0–89)	26 (1 - 89)	47 (4–83)	12 (0–5)	8 (1–36)
low	94 (40%)	23 (34%)	7 (12%)	24 (48%)	40 (73%)
medium	79 (34%)	24 (35%)	15 (25%)	25 (50%)	15 (27%)
high	60 (26%)	21 (31%)	38 (63%)	1 (2%)	0 (0%)
Epithelial proximity (10^{-2} mm):					
average (SD)	18.0 (13)	16.0 (6)	31.0 (13)	24.0 (6)	1.1 (0.2)
median (range)	18 (1–80)	15 (6–32)	31 (5–77)	22 (13–39)	1 (1–2)
low	55 (23%)	0 (0%)	0 (0%)	0 (0%)	55 (100%)
medium	111 (48%)	64 (94%)	17 (28%)	30 (60%)	0 (0%)
high	67 (29%)	4 (6%)	43 (72%)	20 (40%)	0 (0%)
Fibronectin (SS): intensity					
average (SD)	2.1 (1.0)	2.8 (0.5)	1.0 (0.1)	3.0 (0)	1.6 (0.8)
median (range)	3 (1–3)	3 (1–3)	1 (1–2)	3 (3–3)	3 (3–3)
low	96 (41%)	4 (6%)	59 (98%)	0 (0%)	33 (60%)
medium	17 (7%)	3 (4%)	1 (2%)	0 (0%)	13 (24%)
high	120 (52%)	61 (90%)	0 (0%)	50 (100%)	9 (16 %)

pattern					
negative	96 (42%)	4 (6%)	59 (98%)	0 (0%)	33 (60%)
peritumoral	52 (22%)	51 (75%)	0 (0%)	0 (0%)	1 (2 %)
diffuse	85 (36%)	13 (19%)	1 (2%)	50 (100%)	21 (38%)
HA:					
average (SD)	2.4 (0.8)	1.9 (0.9)	2.7 (0.6)	2.4 (0.5)	2.9 (0.4)
median (range)	3 (0–3)	2 (0–3)	3 (1–3)	2 (1–3)	3 (2–3)
low	28 (12%)	22 (32%)	5 (8%)	1 (2%)	0 (0%)
medium	78 (33%)	29 (43%)	10 (17%)	31 (62%)	8 (15%)
high	127 (55%)	17 (25%)	45 (75%)	18 (36%)	47 (85%)
ITGα2 (SS):					
(C) intensity					
average (SD)	3.0 (0.3)	2.7 (0.5)	3.0 (0.1)	3.0 (0.1)	3.0 (0)
median (range)	3 (1–3)	3 (1–3)	3 (2–3)	3 (2–3)	3 (3–3)
low	1 (0%)	1 (1%)	0 (0%)	0 (0%)	0 (0%)
medium	18 (8%)	16 (24%)	1 (2%)	1 (2%)	0 (0%)
high	214 (92%)	51 (75%)	59 (98%)	49 (98%)	55 (100%)
(S) intensity					
average (SD)	1.1 (0.3)	1.2 (0.5)	1.0 (0.2)	1.2 (0.4)	1.0 (0)
median (range)	1 (1–3)	1 (1–3)	1 (1–2)	1 (1–2)	1 (1–1)
low	208 (89%)	53 (78%)	58 (97%)	42 (84%)	55 (100%)
medium	24 (10%)	14 (21%)	2 (3%)	8 (16%)	0 (0%)
high	1 (1%)	1 (1%)	0 (0%)	0 (0%)	0 (0%)
ITGα5 (SS):					
intensity					
average (SD)	2.0 (0.9)	2.7 (0.6)	1.2 (0.5)	2.8 (0.4)	1.6 (0.6)
median (range)	2 (1–3)	3 (1–3)	1 (1–3)	3 (2–3)	2 (1–3)
low	85 (36%)	7 (10%)	52 (87%)	0 (0%)	26 (47%)
medium	47 (20%)	5 (7%)	6 (10%)	10 (20%)	26 (47%)
high	101 (44%)	56 (83%)	2 (3%)	40 (80%)	3 (6%)
pattern					
negative	85 (37%)	7 (10%)	52 (87%)	0 (0%)	26 (47%)
peritumoral	71 (30%)	43 (64%)	2 (3%)	6 (12%)	20 (36%)
diffuse	77 (33%)	18 (26%)	6 (10%)	44 (88%)	9 (16%)
ITGβ1 (SS):					
(C) intensity					
average (SD)	2.0 (0.8)	1.3 (0.5)	2.1 (0.6)	2.1 (0.5)	2.9 (0.3)
median (range)	2 (1–3)	1 (1–3)	2 (1–3)	2 (1–3)	3 (2–3)
low	63 (27%)	52 (78%)	8 (13%)	3 (6%)	0 (0%)
median	94 (40%)	15 (22%)	36 (60%)	39 (78%)	4 (7%)
high	76 (33%)	1 (1%)	16 (27%)	8 (16%)	51 (93%)
(S) intensity					
average (SD)	1.5 (0.6)	1.8 (0.8)	1.2 (0.4)	1.7 (0.5)	1.1 (0.3)
median (range)	1 (1–3)	2 (1–3)	1 (1–2)	2 (1–2)	1 (1–2)
low	143 (61%)	30 (44%)	45 (75%)	16 (32%)	50 (91%)
medium	77 (33%)	25 (37%)	10 (17%)	34 (68%)	5 (9%)
high	13 (6%)	13 (19%)	0 (0%)	0 (0%)	0 (0%)
Ki67 (%):					
average (SD)	27 (15)	23 (13)	13 (7)	31 (12)	42 (12)
median (range)	24 (2–72)	20 (4–61)	13 (2–35)	29 (7–61)	41 (20–72)
low	95 (41%)	33 (49%)	53 (88%)	8 (16%)	1 (2 %)

medium	85 (36%)	25 (37%)	7 (12%)	31 (62%)	22 (40%)
high	53 (23%)	10 (14%)	0 (0%)	11 (22%)	32 (58%)
MMP14 (SS):					
(C) intensity					
average (SD)	1.6 (1.0)	1.5 (0.8)	1.0 (1.0)	2.5 (0.6)	1.8 (0.8)
median (range)	2 (0–3)	1 (0–3)	1 (0–3)	3 (1–3)	2 (0–3)
low	110 (47%)	40 (59%)	46 (77%)	2 (4%)	22 (40%)
medium	73 (31%)	19 (28%)	13 (22%)	19 (38%)	22 (40%)
high	50 (21%)	9 (13%)	1 (2%)	29 (58%)	11 (20%)
(S) intensity					
average (SD)	1.6 (1.1)	2.0 (0.9)	0 (1.0)	2.6 (0.6)	1.8 (0.8)
median (range)	2 (0–3)	2 (0–3)	0 (0–2)	3 (1–3)	2 (0–3)
low	58 (25%)	5 (7%)	49 (82%)	0 (0%)	4 (7%)
median	112 (48%)	42 (62%)	11 (18%)	17 (34%)	42 (76%)
high	63 (27%)	21 (31%)	0 (0%)	33 (66%)	9 (16%)
Stromal cell density (0.05 mm^2):					
average (SD)	52 (32)	68 (27)	38 (17)	83 (29)	21 (9)
median (range)	47 (8–190)	64 (18–135)	37 (13–85)	77 (47–190)	20 (8–51)
low	70 (30%)	4 (6%)	19 (32%)	0 (0%)	47 (85%)
medium	71 (31%)	23 (34%)	34 (57%)	6 (12%)	8 (15%)
high	92 (39%)	41 (60%)	7 (11%)	44 (88%)	0 (0%)
Total collagen (area fraction %):					
average (SD)	17 (16)	17 (10)	36 (13)	7 (9)	4 (3)
median (range)	10 (1–67)	17 (1–46)	35 (12–67)	4 (1–35)	3 (1–11)
low	66 (28%)	4 (6%)	0 (0%)	23 (46%)	39 (71%)
medium	77 (33%)	37 (54%)	3 (5%)	21 (42%)	16 (29%)
high	90 (39%)	27 (40%)	57 (95%)	6 (12%)	0 (0%)

ALDH1, aldehyde dehydrogenase 1; αSMA, α-smooth muscle actin; C, Cancer; Col, collagen; CP, chickenwire pattern; FP, fascicular pattern; HA, hyaluronan; IRS, immunoreactive score; ITG, integrin; MMP14, matrix metalloproteinase 14; P, pattern; PP, periglandular pattern; S, stroma; SD, standard deviation; SS, staining score; TP, tendon-like pattern.

Table S3. Correct and incorrect predictions of morphological patterns based on the full set or subsets of the analyzed features.

**Features (*n*)**	**Correct (%)**	**Incorrect**			
		**PP**	**TP**	**FP**	**CP**
	**(*n* = 233)**	**(*n* = 68)**	**(*n* = 60)**	**(*n* =50)**	**(*n* = 55)**
All (26)	226 (97)	7: 5 as TP<br>2 as FP	0	0	0
Cancer-related (8)	196 (84)	9: 3 as TP<br>6 as FP	6: 5 as PP<br>1 as FP	19: 14 as PP<br>2 as TP<br>3 as CP	3: 1 as PP<br>2 as FP
Stromal (18)	223 (96)	10: 4 as TP<br>6 as FP	0	0	0
ECM (9)	225 (97)	8: 4 as TP	0	0	0

		4 as FP			
Stromal cell-related (8)	206 (88)	24: 6 as TP<br>16 as FP<br>2 as CP	0	3: 3 as PP	0

CP, chicken-wire pattern; ECM, extracellular matrix; FP, fascicular pattern; PP, periglandular pattern; ROI, region of interest; TP, tendon-like pattern.
